# Age Differences in Preferences for Fear-Enhancing Vs. Fear-Reducing News in a Disease Outbreak

**DOI:** 10.3389/fpsyg.2020.589390

**Published:** 2020-12-23

**Authors:** Anthony A. Villalba, Jennifer Tehan Stanley, Jennifer R. Turner, Michael T. Vale, Michelle L. Houston

**Affiliations:** Department of Psychology, University of Akron, Akron, OH, United States

**Keywords:** COVID-19, emotions and aging, positivity effects, fear and aging, behavior change, media engagement, social media, news media

## Abstract

Older adults (OA) prefer positive over negative information in a lab setting, compared to young adults (YA; i.e., *positivity effects*). The extent to which OA avoid negative events or information relevant for their health and safety is not clear. We first investigated age differences in preferences for fear-enhancing vs. fear-reducing news articles during the Ebola Outbreak of 2014. We were able to collect data from 15 YA and 13 OA during this acute health event. Compared to YA, OA were more likely to read the fear-enhancing article, select hand-sanitizer over lip balm, and reported greater fear of Ebola. We further investigated our research question during the COVID-19 pandemic with 164 YA (18–30 years) and 171 OA (60–80 years). Participants responded to an online survey about the COVID-19 pandemic across 13 days during the initial peak of the pandemic in the United States (U.S.). Both YA and OA preferred to read positive over negative news about the coronavirus, but OA were even more likely than YA to prefer the positive news article. No age differences in the fear of contraction were found, but OA engaged in more health-protective behaviors compared to YA. Although OA may not always report greater fear than YA or seek out negative information related to a health concern, they still engage in protective health behaviors. Thus, although *positivity effects* were observed in attention and emotional reports (in the COVID-19 study), OA still modified their behaviors more than YA (giveaway in both studies, and health-protective behavior change in the COVID-19 study), suggesting that positivity effects did not hamper OA ability to respond to a health crisis.

## Introduction

Few emotions exemplify evolutionary fitness as clearly as fear. Fear is primarily associated with inhibition (e.g., withdrawal) or avoidance (e.g., fleeing) behaviors, which can provide health-protective features by elevating concerns for personal safety (De Gelder et al., 2004; Carver, 2006). This practical argument for the protective nature of fear forms the foundational crux of the *negativity bias* that has been documented extensively in young adults (YA) and children (Baumeister et al., 2001; Vaish et al., 2008; LoBue, 2009). Across many samples and situations, YA consistently demonstrate a preference for attending to and remembering negative information, compared to positive, and this preference has implications for adaptive success theoretically (e.g., increased longevity; Baumeister et al., 2001; Vaish et al., 2008). In comparison, research on attentional deployment and memory in mid-life and later-life conveys a different story: older adults (OA), when compared with YA, prefer to engage with and remember more positive information over negative. Termed the *positivity effect*, this result has been replicated in numerous laboratory studies with diverse explanations varying from neuronal decline with age to motivations for enhanced well-being (Cacioppo et al., 2011; Reed et al., 2014; Carstensen and DeLiema, 2018). However, one question that remains is the extent to which these findings translate out of the lab and whether researchers can detect positivity effects using a major real-life event (e.g., a major health-related event). Similar to Ford et al. (2018), who used a highly negative public event (i.e., Boston Marathon bombings), we sought to determine whether positivity effects would emerge in the context of a disease outbreak or pandemic. Specifically, will age differences in attention to information regarding fear-provoking stimuli still be found in the context of a public health crisis such as a disease outbreak or pandemic?

Past researchers have found that OA, compared to YA, demonstrate overall lower levels of fear and worry across many domains (e.g., environmental concerns, phobias, etc.); however, the opposite pattern was observed when the stimuli involved health risks and concerns (Powers et al., 1992; Teachman and Gordon, 2009). Further, in a laboratory study, OA selectively attended to negative health-related information in a manner that mitigated negative mood outcomes, but promoted positive health behaviors, suggesting that OA will engage with negative stimuli if it serves a health-protecting benefit (Isaacowitz and Choi, 2012). Most of the studies investigating age differences in fearful responses have been hampered by the difficulty of inducing high levels of fear in the laboratory (e.g., Stanley and Isaacowitz, 2015). Therefore, the present study capitalized on a naturally occurring fear event to investigate age differences in preferences for positive information, levels of fear, and health-related behavior change during a health pandemic.

Two recent health-related fear events include the Ebola outbreak of 2014, and the ongoing COVID-19 pandemic. For both events, these viruses led to rapid changes in public policy, health and safety, and media coverage; the Centers for Disease Control and Prevention (CDC) issued travel warnings and bulletins regarding quarantines and at-risk populations (Centers for Disease Control and Prevention, 2020). The Ebola virus disease is a hemorrhagic fever with an average case fatality rate of 50% (World Health Organization, 2020). The novel coronavirus causes a respiratory disease, COVID-19, with a current estimated case fatality rate in the United States (U.S.) of 3.5% (this rate is subject to change as we learn more about the virus; Johns Hopkins University: Coronavirus Resource Center, n.d.). Both viruses have relatively long incubation periods (2–21 days for Ebola and 2–14 days for COVID-19). In comparison to the Ebola Outbreak of 2014, which resulted in 11 people being treated in the U.S. with one fatality, the overall health impact and loss of life from COVID-19 is much worse in the U.S.: by July 25, 2020, the number of U.S. deaths surpassed 143,000 individuals. Further, regarding COVID-19, OA are at heightened risk for severe illness and death (Centers for Disease Control and Prevention, 2020). COVID-19 is an airborne virus that can be contracted through near/close contact to someone with the virus, mainly through inhaling respiratory droplets (Centers for Disease Control and Prevention, 2020). Ebola is contracted through blood/bodily fluid contact with someone who has Ebola, or contact with contaminated food or an infected animal (Centers for Disease Control and Prevention, 2020). Health providers who care for those who have contracted Ebola are at the greatest risk for contraction.

While the COVID-19 pandemic is affecting people globally in a number of ways, this paper will focus on perceptions and fear of COVID-19 in the U.S. among YA and OA during the implementation of stay-at-home orders across the country. During the 13 days we collected data, the number of deaths in the U.S. increased from just over 56,000 deaths to nearly 81,000 deaths. Many states implemented systematic shutdowns of schools, businesses, and organizations, following CDC guidelines that recommended social distancing, wearing masks, and handwashing (Centers for Disease Control and Prevention, 2020). For most states, the stay-at-home orders began in late March, 2020 (first statewide order was March 19, 2020) and extended until late April, 2020 with the intention of protecting those most at risk (i.e., older adults) and “flattening the curve” so that the hospitals would not exceed capacity to care for those affected (Centers for Disease Control and Prevention, 2020). This paper will focus on the perceptions and fear of both Ebola and COVID-19. These events differ in the type of disease, contraction, and death rates in the United States, disease transmission, and types of at-risk populations. However, both are major health-related events, with Ebola showing major importance in the local area of data collection, and provide an opportunity to investigate age differences in fear-seeking vs. fear-reducing behaviors. We first investigated age differences in attention to fearful information in the context of the Ebola outbreak in 2014, and then built upon that initial study, correcting for design limitations and power issues, to further investigate the same research questions during the COVID-19 pandemic.

### Age Differences in Fear

Worry and fear manifest differently for YA vs. OA (Carstensen, 1988; Kogan and Edelstein, 2004). For instance, while OA experience less intense and fewer fears compared to YA, OA report more worry for world issues (Kirkpatrick, 1984; Hunt et al., 2003). YA not only worry more overall, but their worries are mostly about financial and social issues (Powers et al., 1992). However, OA report greater fear of isolation/separation from loved ones, which is especially relevant during the COVID-19 pandemic (Kogan and Edelstein, 2004; Morrow-Howell et al., 2020). U.S. guidelines require that anyone who has contracted or been in proximity with someone who contracted COVID-19 be quarantined, so OA may have more fear of contracting the virus due to the separation components.

Older adults also report more worries about their general health compared to YA (Hunt et al., 2003). The authors speculate that OA may use the feelings of fear or worry as a means for problem-solving, leading to more efficient problem-solving than YA. This may explain why OA engage *less* in information seeking but engage *more* in behavioral change for health-related stimuli (Isaacowitz and Choi, 2012). However, during the Severe Acute Respiratory (SARS) epidemic in Hong Kong, worrying positively predicted health behavior changes for OA (Lau et al., 2005). Therefore, it is unclear how fear or worry promotes or inhibits behavior change to prevent falling ill, specifically for OA.

In a more recent study examining COVID-19, OA reported higher perceived risks associated with coronavirus when compared to YA (Barber and Kim, 2020). These authors found that the relationship between worry and behavioral changes were observed across all age groups, however, older adult men were the least worried about COVID-19, and they also implemented the fewest number of behavior changes compared to the other age and gender groups. This led the authors to report that emotional responses to COVID-19 are successful predictors of behavioral change responses. The authors also found lower worry in OA relative to YA. It should be noted that our hypotheses for both studies predicted the opposite results (OA would report greater fear and behavior change than YA). Our reasoning was that OA would be more fearful than YA because they would feel more vulnerable than YA, perhaps because their immune system is weaker making it more difficult for them to fight off the disease. There are a few reasons for this contradiction between the Kim and Barber (2020) study findings and our hypotheses. First, the Kim and Barber study was not published when we made our predictions, so we could not use these data to inform our predictions. Second, Kim and Barber collected data in the same month that COVID-19 was first declared a pandemic by the World Health Organization (March, 2020), while we collected data a month later, in late April and early May, 2020. It is possible that the level of worry and fear increased from the earliest days of the pandemic, after a month of increasing cases and deaths from the virus. Finally, while there is a lot of overlap between Kim and Barber’s COVID-19 worries composite measure and our pandemic-related fear composite measure, our measure focuses on health-related fears only (fear of contraction, fear of loved ones contracting the virus), while Kim and Barber’s measure also includes worries about the economy and disruptions to one’s lifestyle. As the literature grows in examining the role of fear or worry in health-related behavioral changes, we aim to provide more information on the role of age differences in emotional functioning in predicting health-related behaviors.

### Age Differences in Attention to Negative Information

The impetus for the current investigation was to examine whether positivity effects emerge in the context of a naturally occurring event relevant to health and safety. Other studies have explored positivity effects in the same manner (Ready et al., 2007; Schryer and Ross, 2014). In a laboratory study investigating a similar question, OA exhibited positivity effects by showing less engagement with negative content about skin cancer compared to YA, but also engaged in more health-protective behaviors than YA after exposure to the skin cancer information (Isaacowitz and Choi, 2012). Although OA may not have dwelled on the negative information, they still seemed to take the information seriously, resulting in more behavior change to avoid negative health outcomes. Isaacowitz and Choi (2012) interpreted these findings as an efficient age-related strategy that OA use to extract important information without negatively impacting their moods. In contrast, YA may be consuming too much negative information, resulting in mood disruption, which affects their ability to successfully engage in protective behavior change.

Our main research question is whether positivity effects will be evident when adults are living through a disease outbreak or pandemic. *Socioemotional Selectivity Theory* (SST) posits that OA prioritize emotional goals (i.e., well-being) and thus may be more likely than YA to avoid negative health information (Carstensen et al., 1999). Additionally, goal preferences may not be consistent across all contextual factors of decision making (Löckenhoff and Carstensen, 2004). For example, OA may be more concerned about maintaining a positive mood and therefore avoid negative health information regarding a serious health threat. In one study, OA attended to and remembered a greater amount of positive vs. negative information about physicians and health care plans when compared to YA, and this was in part due to a limited time perspective (Löckenhoff and Carstensen, 2007). However, Reed and Carstensen (2012) explain that positivity effects do not always occur. For example, when OA are in contexts that require situation-specific goals, or if the prioritizing of emotional goals is associated with significant risks, then positivity effects may not be observed. We consider the present study a test of this claim. Furthermore, the *Strength and Vulnerability Integration* (SAVI) model also describes why OA avoid, or attempt to reduce, exposure to material that causes emotional distress (Charles, 2010). The rationale for this avoidance, according to the SAVI model, is that OA, when experiencing high levels of emotional arousal, spend more time in the high arousal state and take longer to return to baseline, when compared to YA. Therefore, avoiding high arousal material or events is advantageous for OA due to the increased physiological toll these emotional states can render on OA.

In contrast to the theories presented above, OA tend to watch news media more than YA, despite news being mostly negative (Mares and Woodard, 2006; Mitchell et al., 2016). The violence portrayed in TV news media elicits primarily negative emotions such as fear, anger, and contempt (Unz et al., 2008). In addition, exposure to news media is related to increased negative stress (McNaughton-Cassill, 2001). Thus, it is important to understand more about how media engagement affects age differences in the relationship between preferences for affective material and behavior change.

## Current Studies

### Study 1 Age Differences in Fear During the Ebola Outbreak: A Feasibility Study

In 2014, during the Ebola outbreak in West Africa, we conducted a study to examine whether OA would attend to fearful health information tied to an actual current health threat (i.e., Ebola). We had a unique local situation because a nurse who was exposed to Ebola in Dallas, Texas, visited Akron, Ohio to plan her wedding in October 2014, 2 days before she was diagnosed with Ebola after returning to Texas. Northeast Ohio had extensive media coverage of this situation (e.g., Gittens and Connor, 2014). On October 23, 2014, we started data collection, 8 days after the nurse was diagnosed with Ebola, and concluded data collection on December 5, 2014. The framework and preliminary results from this feasibility study were extended to the current COVID-19 pandemic for the main study.

### Hypotheses

We first hypothesized that *OA would attend to fearful health-related information about Ebola more compared to YA* (Hypothesis 1A). The rationale for this hypothesis was that OA would be motivated to increase their knowledge about the Ebola outbreak so they could better avoid negative health outcomes. This would be an example of a task-relevant goal overriding the chronic goal that theoretically drives the positivity effect (Reed and Carstensen, 2012). For the next hypothesis, we predicted that *OA would be more likely, relative to YA, to select hand sanitizer over lip balm in a giveaway directly after the study* (Hypothesis 1B). For this giveaway, we wanted to show that OA would be more likely to participate in health-protective behaviors when compared to their younger counterparts, consistent with Isaacowitz and Choi (2012). Next, *we expected that OA would fear contracting Ebola more than would YA* (Hypothesis 2), because OA might consider themselves more vulnerable to health threats than do YA. Lastly, we hypothesized that *OA would show greater health-related change in behaviors compared to YA* (Hypothesis 3) as a result of OA greater fear of contracting Ebola.

### Methods

#### Participants

**Figure 1:**
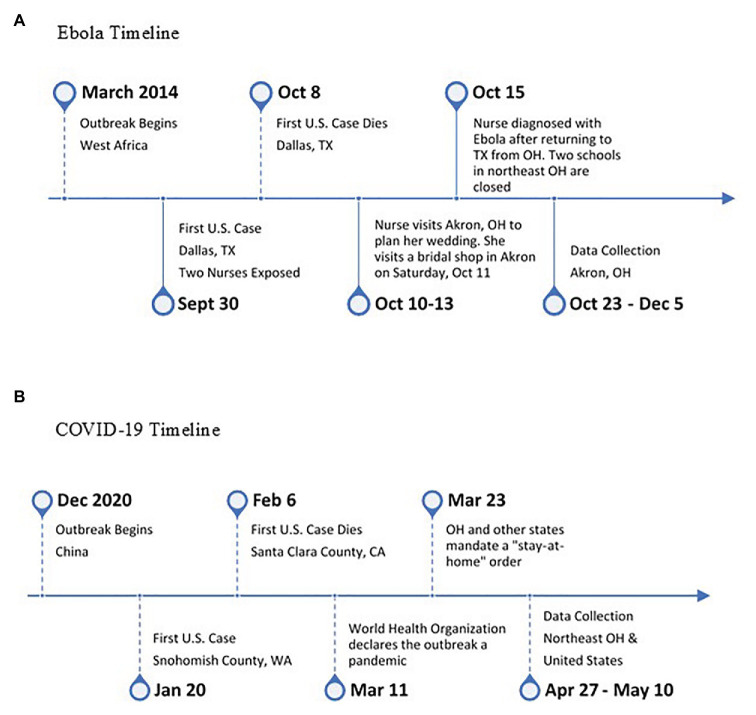
Major event timelines for both studies. **(A)** Ebola timeline and **(B)** COVID-19 timeline.

Data were collected from 28 participants through psychology classes for young adults and the local community for older adults. We were able to collect data from 15 YA (ages 18–30, *M* = 21.20, *SD* = 5.28; 33% female) and 13 OA (ages 60–80, *M* = 68.69, *SD* = 4.77; 54% female) in the immediate weeks following the visit from the nurse (see Figure 1A for the Ebola timeline).

#### Design and Procedure

Participants came to the lab to participate in a different study and were asked to wait 5 min in a waiting room. Here, they were told they could browse news articles open on the computer screen while they waited. They could choose between reading either a fear-enhancing article, titled, *Why Ebola is so Dangerous*, or a fear-reducing article, titled *Reasons to Calm Down About Ebola*. The presentation order of the article titles on the screen (left or right) was counterbalanced across participants. The fear-reducing article source was from the Wallstreet Journal (939 words), and the fear-enhancing article source was from the BBC-News-Africa (1,137 words). The website article sources were not available to participants and not specific to the local area. The articles were equated on positive and negative affective words as determined by Linguistic Inquiry Word Count software (Pennebaker et al., 2015). The fear-enhancing article contained one image, while the fear-reducing article had no images. After 5 min, the researcher came back into the room to tell the participant that they were ready to start the study. We did not measure the amount of time participants spent reading the articles; we only collected data on which article was selected during the waiting period. The participants were offered a “give-away” of either hand sanitizer or lip balm as a thank you for their patience in waiting for the study to begin. The intervening studies individuals participated in before responding to their feelings/behaviors related to Ebola consisted of studies on social judgments (i.e., emotion perception tasks, deceit detection tasks) or emotion regulation knowledge. None of these intervening studies intentionally induced fear or anxiety or relate to Ebola or disease. Each intervening study took an hour or less to complete. After participating in the other study, participants responded to four items assessing their feelings about contracting Ebola and behaviors they have changed in response to the Ebola outbreak. After completing the questionnaire, the researcher explained that their behaviors in the waiting room were actually part of a study on the Ebola outbreak, in combination with their responses to the Fear of Ebola Questionnaire, and asked for their consent to use their data. All participants provided informed consent and the study was IRB approved. As part of the debriefing, participants received a handout with information on Ebola from the Centers for Disease Control.

#### Measures

##### Ebola-Related Fear of Contraction

Participants responded to four items assessing their fear of contracting Ebola: “*How often in the past week…Did you fear that you could contract Ebola? Did you fear one of your loved ones could contract Ebola? Did you think about Ebola?*” were rated from 1 (*Not at all*) to 5 (*Extremely often*) and “*How afraid are you of contracting Ebola?*” was rated from 1 (*Not at all*) to 5 (*Extremely*). The reliability of these four items on the Ebola-Related Fear of Contraction Questionnaire was *α* =0.79.

##### Ebola-Related Behavior Change

To assess whether participants changed their behaviors in accordance with the Ebola outbreak, we asked them to respond with either a “Yes” or “No” to four items: “*Since the Ebola outbreak, have you changed any habits? Washing Hands (yes/no), Visiting Stores (yes/no), Air Travel (yes/no), Attending Events (yes/no)*”. The reliability of these four items on the Ebola-Related Behavior Change Questionnaire was poor, *α* =0.53. Ebola-related behavior change scores were calculated by summing the total amount of yes responses across all four items.

### Results

#### Age Differences in Article and Giveaway Selection (Hypotheses 1A and 1B)

To examine whether there were age differences in the article and giveaway selections, we conducted two Pearson Chi-square tests. Some participants selected neither article (YA: *n* = 3, OA: *n* = 4), did not accept a giveaway item (YA: *n* = 1, OA: *n* = 2), or accepted both giveaway items (OA: *n* = 1). Thus, these participants were not included in the respective analyses, leaving 12 YA (*M* = 20.83 years, *SD* = 3.19, 58% male) and nine OA (*M* = 66.89 years, *SD* = 2.71, 56% female) for testing the article selection hypothesis, and 15 YA (*M* = 20.53 years, *SD* = 3.02, 66% male) and 12 OA (*M* = 68.83 years, *SD* = 4.95, 50% female) for the giveaway selection hypothesis.

**Figure 2:**
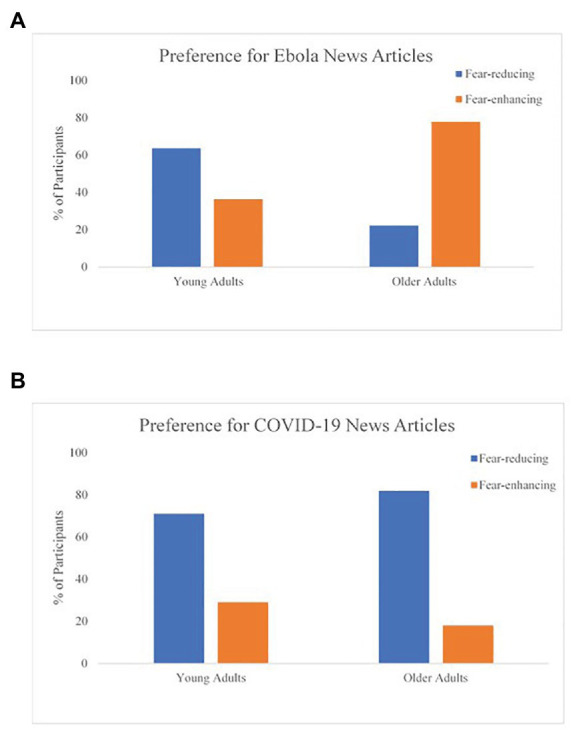
Age group comparisons for the preference of news article for both studies. **(A)** Preference for Ebola news articles and **(B)** preference for COVID-19 news articles.

**Figure 3:**
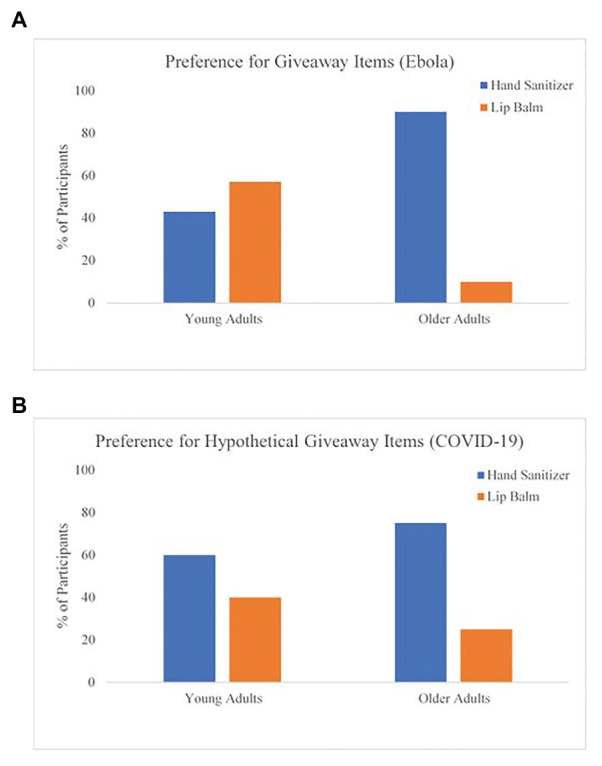
Age group comparisons for the giveaway items for both studies. **(A)** Preference for giveaway items (Ebola) and **(B)** preference for hypothetical giveaway items (COVID-19).

Consistent with our predictions, OA were more likely to choose the fear-enhancing article, while YA were more likely to choose the fear-reducing article, *χ*^2^(2) = 4.07, one-tailed *p* = 0.02, Ф = 0.44. In addition, OA were more likely than YA to choose hand sanitizer over lip balm, *χ*^2^(2) = 7.27, one-tailed *p* = 0.03, Ф = 0.52. Together, this suggests that OA attended to the fear-enhancing material and selected a health-relevant token more than YA, which aligned with our hypotheses (results are depicted in Figures 2A, 3A).

#### Age Differences in the Fear of Ebola (Hypothesis 2)

**Figure 4:**
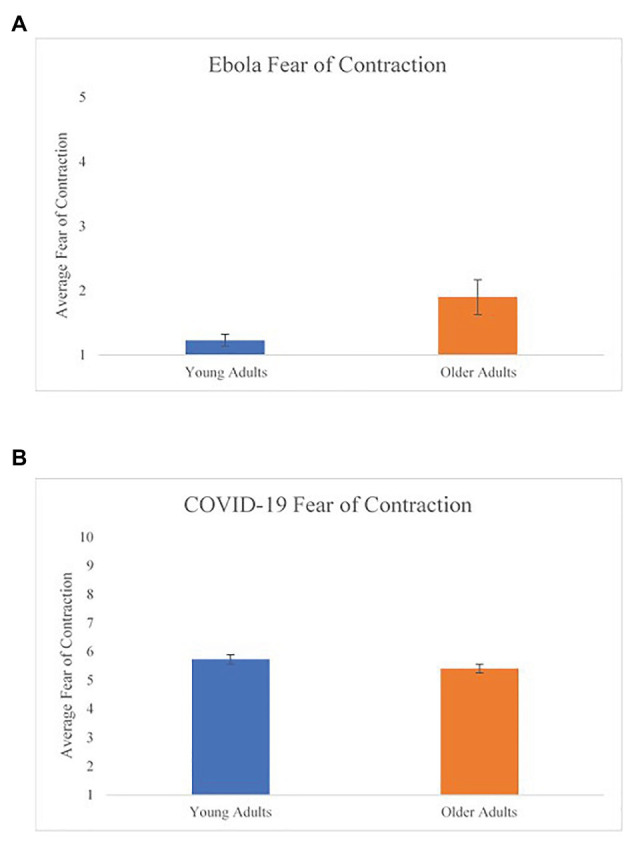
Age differences in fear of contraction for both studies. **(A)** Ebola fear of contraction and **(B)** COVID-19 fear of contraction. Error bars represent the standard error for each age group.

To test for age differences in the fear of contracting Ebola, we conducted an independent-samples *t*-test. OA (*M* = 1.91, *SD* = 0.98) reported greater fear of contracting Ebola compared to YA (*M* = 1.23, *SD* = 0.36), *t*(26) = 2.47, one-tailed *p* = 0.02, Cohen’s *d* = 0.91. Additional analyses revealed that article selection did not predict fear of contraction [*F*(1,26) = 0.74, *p* = 0.40, *R*^2^ = 0.03]. It is also important to note that older adults’ average fear of Ebola did not exceed two on a five-point scale, suggesting that neither age group reported *high* fear of contracting Ebola, on average. Nevertheless, the pattern of results are consistent with our hypothesis (Figure 4A).

#### Age Differences in Ebola-Related Behavior Change (Hypothesis 3)

**Figure 5:**
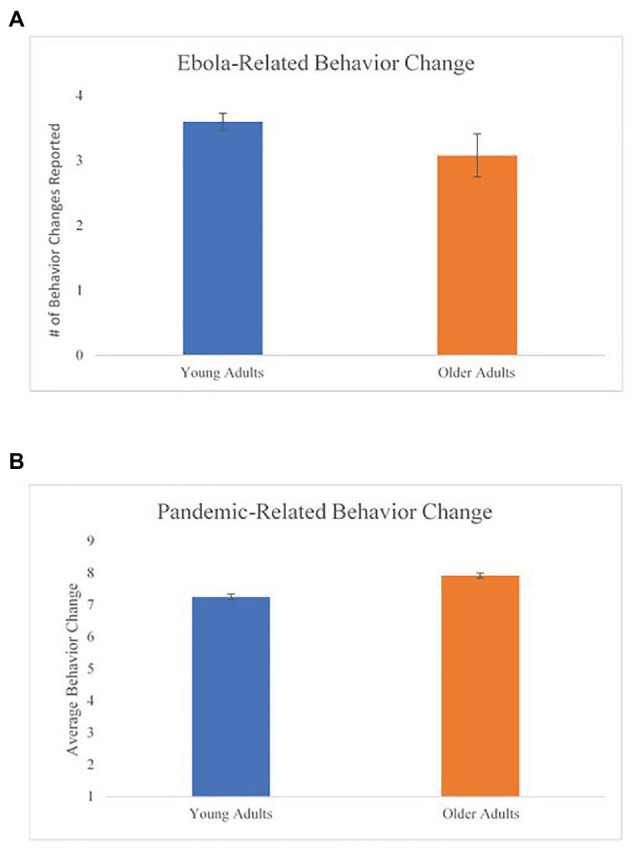
Age differences in health-related behavior change for both studies. **(A)** Ebola-related behavior change and **(B)** pandemic-related behavior change. Error bars represent the standard error for each age group.

An independent-samples *t*-test comparing YA and OA on the Ebola-related behavior change variable was not significant *t*(26) = 1.56, one-tailed *p* = 0.13. Results indicate that YA and OA reported a similar number of behavior changes in response to the Ebola outbreak (YA: *M* = 3.60, *SD* = 0.51; OA: *M* = 3.08, *SD* = 1.19). These results were not consistent with our hypothesis (Figure 5A).

### Discussion

Although the sample size was small, we were interested in the pattern of findings to inform the design of the COVID-19 study. We found that OA *will* interact with negative information when it is relevant to their health and safety. OA reported a greater fear of contracting Ebola compared to YA, and also engaged in greater health-protective behaviors by selecting hand sanitizer over lip balm comparatively. Overall, participants in both age groups reported low levels of fear of contracting Ebola. This study suggested that investigating behaviors in YA and OA in a naturally occurring fearful situation/event may be a fruitful avenue for future research.

However, these are tentative results given the small sample size and lack of statistical power. We were able to modify our measures to better capture age differences in disease-related behavior change during the COVID-19 pandemic. For example, we modified the disease-related behavior change measure from a dichotomous scale to a continuous scale to increase the sensitivity of our measurement (MacCallum et al., 2002). We also wanted to assess whether media engagement would relate to age differences in fear and attention to negative health information. In addition, in this first study participants were able to select none or all items in our article and giveaway selections, which reduced our sample size for examining preferences. Finally, we wanted to test whether these findings would replicate during a different health-related event, especially an event where OA are an at-risk population. Thus, we continued to explore our research questions in the next study, while enhancing our materials and design, by examining age differences in fear related to the COVID-19 pandemic and assessing how media engagement relates to pandemic-related fear.

### Study 2 Age Differences in Fear During the Covid-19 Pandemic

Older adults have been shown to avoid high arousal and negative information, perhaps as a means to promote overall well-being. But the question is, do these preferences persist when the negative information is related to a serious health event? Furthermore, how do preferences in information-processing relate to protective behavior change? Study 1 showed that OA will engage in fear-enhancing material, yet, still engage in disease-related behavior change by selecting health-protective items when given the option (i.e., hand sanitizer over lip balm). However, do these findings replicate for health threats that are more relevant for OA than YA? Specifically, regarding COVID-19, OA are part of a high-risk population and avoidance of health-related information may be detrimental for their health and safety, or even worse, result in death. Consequently, it is vital to understand whether OA are receiving this important information and changing their protective health behaviors accordingly, especially if OA are part of an at-risk population. If they are not doing so, future intervention research must be conducted to attenuate these biases to help avoid negative consequences for the OA population. Therefore, the current investigation was meant to test whether OA attend to fearful health information tied to an actual current health threat.

### Hypotheses

We pre-registered most of our hypotheses on the Open Science Framework.^1^,^2^ First, based on the results of the Ebola pilot study, we hypothesized that *OA would be more likely than YA to select the fear-enhancing article about COVID-19* (Hypothesis 1A). The rationale for this prediction was that OA may attend to negative information more than YA in order to increase their knowledge so they can avoid negative health outcomes, especially given OA are more vulnerable to the coronavirus. Next, consistent with the results of our pilot study, we expected *OA to be more likely than YA to select the hand sanitizer (vs. the lip balm) in our hypothetical give-away* (Hypothesis 1B). For Hypothesis 2, we predicted that *OA would report greater fear of contracting the coronavirus than YA*. We also expected *OA to report greater change in virus-related health behaviors than YA* (Hypothesis 3). Hypotheses 4 and 5 relate to media engagement, in that we expected *those participants who spent less time engaging in media outlets (i.e., news and social media) would show less fear of contracting the coronavirus* (Hypothesis 4), and that *news media engagement would moderate the relationship between age and fear of contraction* (Hypothesis 5), such that the positive relationship between age and fear of contraction would be reduced for those who engage less with media.

### Methods

#### Participants

A power analysis indicated we would need at least 108 participants to detect a medium effect from a chi-square test comparing whether young or older adults are more likely to select one choice over the other (with power = 0.80 and alpha = 0.05). The study was approved by the IRB and participants provided informed consent. Data were collected from 365 participants through an online survey. Data from 28 participants were removed from analyses (~8%) due to invalid responses (*n* = 2), failure to meet the age criteria (*n* = 22), or less than 10% of the survey completed (*n* = 4). This resulted in 337 participants for data analysis. All variables in the analyses were checked for normal distribution violations (i.e., skewness and kurtosis) and outliers. We found that the pandemic-related behavior change variable had skewness and kurtosis issues due to two YA outliers (one male and one female) who were >3.29 SDs from the mean in a negative direction (Tabachnick and Fidell, 2019). Once these participants were removed from the data set, we found that this variable was normally distributed. All other variables exhibited adequate normality and showed no apparent outliers. Thus, our final data analysis sample consisted of 335 participants. All inferential statistics are two-tailed. In addition, there were some participants who only completed part of the survey (*n* = 37, ~10% of the original data set). These participants failed to respond to the final question in the survey: the hypothetical giveaway. However, these participants responded to the rest of the key items, and we found no significant differences on these variables between those who did versus did not complete the study; therefore, we included them in all analyses except the hypothetical giveaway analysis, resulting in 298 participants for Hypothesis 1B.

Overall, 164 YA (ages 18–30 years, *M* = 23.72 years, *SD* = 3.56) and 171 OA (ages 60–80 years, *M* = 68.41 years, *SD* = 4.86) participated in this study and met our inclusion criteria. The sample was comprised of mostly female (YA: 77%, OA: 70%), non-Hispanic (YA: 88%, OA: 97%), Caucasian (YA: 87%, OA: 96%) participants. Unsurprisingly, given that the YA are still in the process of completing their education, the OA (*M* = 16.24, *SD* = 2.62) reported completing more years of formal education than the YA [*M* = 14.82, *SD* = 2.32; *t*(333) = 5.24, *p* < 0.001]. We also collected background variables such as income, whether one was an essential worker, whether one worked from home, political affiliation, and state of residence. See Table 1 for summary statistics and age differences in background characteristics. All characteristics with significant age group differences were included as covariates in subsequent analyses.

**Table 1:** Background characteristics for young adults (YA) and older adults (OA).

Variable	Young adults	Older adults	Statistic	*p*
	*n* = 164	*n* = 171		
Gender				
Male	36 (22%)	51 (30%)		
Female	127 (77%)	119 (70%)	*χ*^2^(2) = 2.70	0.26
Racial identity				
African American/Black	8 (5%)	0 (0%)		
Asian American/Pacific Islander	9 (6%)	3 (2%)		
European American/White	142 (87%)	164 (96%)		
Other	5 (3%)	4 (2%)	*χ*^2^(5) = 16.24	<0.01
Ethnic identity				
Hispanic	8 (5%)	1 (1%)		
Non-hispanic	144 (9%)	166 (97%)		
Other	12 (7%)	4 (2%)	*χ*^2^(2) = 10.86	<0.01
Recruitment source				
AMT	54 (33%)	62 (36%)		
Social media/Database	110 (67%)	109 (64%)	*χ*^2^(2) = 0.41	0.52
Essential worker				
Yes	62 (38%)	22 (13%)		
No	102 (62%)	149 (87%)	*χ*^2^(2) = 27.71	<0.01
At home worker				
Yes	124 (66%)	151 (88%)		
No	40 (24%)	20 (12%)	*χ*^2^(2) = 9.18	< 0.01
Political affiliation				
Democrat	75 (46%)	84 (49%)		
Republican	34 (21%)	47 (27%)		
Independent	46 (28%)	37 (22%)		
Other	9 (5%)	3 (2%)	*χ*^2^(2) = 6.43	0.09
Ohio resident				
Yes	88 (54%)	84 (49%)		
No	76 (46%)	87 (51%)	*χ*^2^(2) = 0.69	0.41
Age	23.72 (3.56)	68.41 (4.86)	*t*(333) = −95.64	<0.001
Health	3.72 (0.92)	3.64 (0.93)	*t*(333) = 0.81	0.42
Education	14.82 (2.32)	16.24 (2.62)	*t*(333) = 5.24	<0.001
Income	3.90 (2.28)	4.73 (1.97)	*t*(333) = −3.54	<0.001

Gender, racial/ethnic identity, recruitment source, essential worker, at home worker, political affiliations, and whether the participants lived in Ohio or another state were assessed using chi-square analyses. Age, health, and education were analyzed using an independent samples *t*-test. AMT: Amazon’s Mechanical Turk; Database: participants recruited through the lab database for older adults. For the variables age, health, income, and education, the mean and SD [i.e., *M* (*SD*)] was reported for the YA and OA, rather than the subsample size and percentage [i.e., *n* (%)].

#### Design and Procedure

Participants were recruited through social media (i.e., Facebook), an existing database of older adult volunteers, and Amazon’s Mechanical Turk (AMT) using panels in TurkPrime.com (Chandler et al., 2019). Participants recruited through Facebook were mostly friends, family members, and acquaintances of the authors. The recruitment post was shared by others, which may have resulted in the recruitment of the friends, family members, and acquaintances of the researchers’ social networks. Participants recruited *via* Facebook or from our existing database of volunteers were not compensated. Participants were asked to answer a 20-min questionnaire related to their thoughts, feelings, and behaviors in response to the COVID-19 pandemic. Materials can be found on the Open Science Framework.^3^ Inclusionary criteria for this study involved falling within the age ranges of 18–30 or 60–80 years, being a native English speaker, and residing in the United States. Only the participants recruited through AMT received a monetary incentive ($2). Thirty-seven percent of the sample (*N* = 110) was recruited through AMT, while 63% was recruited through social media or the existing pool of volunteers.

We examined whether there were any differences between recruitment sources on the main dependent variables of interest. We found a significant group difference for the frequency of engaging in social media, *t*(332) = 3.77, *p* < 0.001, with the non-AMT group (*M* = 4.04, *SD* = 1.12) engaging in social media more frequently than the AMT group (*M* = 3.51, *SD* = 1.37). All other variables of interest did not differ by recruitment source. We also did not find any Age × Date of Completion (those participants who completed the survey before or after the stay-at-home order was lifted in Ohio on 05/01/2020) interactions for fear of contraction or pandemic-related behavior change outcome variables.

Data were collected across 13 days between 04/27/20 and 05/10/20 (see Figure 1B for COVID-19 timeline). During data collection, the United States had experienced just over 56,000 deaths due to COVID-19 and just over 1.3 million confirmed cases (Centers for Disease Control and Prevention, 2020). In the state of Ohio during the time of data collection, there were 24,801 confirmed and probable cases and 1,341 deaths due to the coronavirus (Centers for Disease Control and Prevention, 2020). In Ohio, participants were under a stay-at-home order through April 30th, which included a ban on mass gatherings and a mandatory two-week isolation period for anyone entering the state. The stay-at-home order was later transitioned to a safe-at-home order on 05/01/20, which allowed nonessential businesses to reopen and gatherings of 10 or fewer people. Data for this project can be found on the Open Science Framework.^4^

#### Measures

##### Pandemic-Related Fear of Contraction

Similar to the pilot study during the Ebola outbreak, participants responded to four items regarding their fear of contracting the coronavirus. The items were as follows: *How often in the last week did you fear that you would contract the coronavirus?*, *How often in the past week did you fear one of your loved ones would contract the coronavirus?*, *How often in the last week did you think about the coronavirus?*, and *How afraid are you of contracting the virus?* Participants responded to the first three items using a Likert-type scale ranging from 1 (*Not at all*) to 10 (*Extremely often*), and 1 (*Not at all*) to 10 (*Extremely*) for the last item. The reliability of this scale was acceptable (*α* = 0.80). A composite *fear* score was created by averaging the four items.

##### Pandemic-Related Behavior Change

Similar to the pilot study, participants responded to six items regarding health-related behaviors they have changed in response to the pandemic. Participants rated their change in behaviors on the frequency of washing hands, duration of washing hands, frequency of visiting stores, amount of time spent inside stores, plans to travel by airplane, and frequency of leaving their house/property since the COVID-19 pandemic, on a Likert-type scale between 1 (*Extremely Decreased*) and 9 (*Extremely Increased*). We reverse-scored the last four items so that those who both increased frequency/duration of handwashing, and decreased the frequency of public engagement, had higher scores on the behavior change variable (higher scores indicating greater protective behaviors). This scale had acceptable reliability (*α* = 0.78). We created a composite score for this scale by averaging the six items.

##### Fear-Enhancing Vs. Fear-Reducing Article Preference

Similar to the pilot study, participants were asked to choose between reading an article titled, *There’s More Bad News on the Long-Term Effects of the Coronavirus* and *10 Positive Updates on the COVID-19 Outbreaks from Around the World*. The verbatim wording for this item was, *Both articles below are about the COVID-19 outbreak. Choose the article you would prefer to read*. Participants did not actually read these articles, but this item was intended to investigate age differences in selecting an article that was either fear-enhancing or fear-reducing. The article selection was presented directly after the demographic questions, as our first primary measure. We counterbalanced the order in which the article headlines appeared (first or second) across participants and found no order effects.

##### Hypothetical Gift Giveaway

Similar to the pilot study, we formulated a hypothetical giveaway of either hand sanitizer or lip balm. At the end of the study, we asked participants, *If we did this study in person and offered a “giveaway” to participants for completing the survey, hypothetically speaking, which would you select*? Participants selected either lip balm or hand sanitizer, with the order of presentation counterbalanced across participants (first or second). We found no order effects for this item. This item was meant to examine age differences in preventative health-related behaviors in the context of the COVID-19 pandemic.

##### Media Engagement

We were also interested in potential age differences in engagement with the news media, and how that engagement might interact with pandemic-related feelings of fear or behavior change. We included two items that asked participants how often they engaged with social media and news media outlets. Participants were asked to rate on a Likert-type scale of 1 (*Never*) to 5 (*Always*): “…*the frequency that you engage in each activity, since the coronavirus outbreak in the United States: (1) Access social media or other forms of independently-generated media (e.g., Facebook, Twitter, Reddit, forums, blogs, etc.), and (2) Watch, read, or listen to the news media (e.g., local or national news channel, radio station, newspaper articles, news websites, etc.)*”. We used non-parametric tests to analyze responses to the media engagement items because they are one-item inventories with ordinal responses.

#### Results

##### Age Differences in Article and Giveaway Selection (Hypotheses 1A and 1B)

To test for age differences in article and giveaway selections, we conducted two Pearson Chi-square tests. While the majority of participants in both age groups preferred the fear-reducing article titled, *10 Positive Updates on the COVID-19 Outbreaks from Around the World* (YA: 71%, OA: 82%), the OA were significantly *more* likely than YA to choose the fear-reducing article, *χ*^2^(1, *N* = 333) = 6.02, *p* = 0.02, Ф = −0.13. These results do not support our prediction for Hypothesis 1A and are inconsistent with the results of our pilot study. Instead, these findings align with the positivity effects literature and show that OA preferred positive materials more than YA (Figure 2B). Given the findings that YA preferred the fear-reducing article vs. the fear-enhancing article and that social media engagement for this group appeared to be quite high (*M* = 4.35, *SD* = 0.83), we investigated whether social media engagement significantly predicted article selection for the YA group. It is possible that greater social media engagement could have led to an overexposure of negative media coverage, which in turn might render positive news articles more enticing. To examine this question, we conducted a logistic regression using Hayes’ PROCESS to investigate whether the relationship between social media engagement and article selection was moderated by age group, using the covariates from Table 1. The interaction effect was not significant [*β* = −0.14, *SE* = 0.28, 95% CI (−0.692, 0.413)].

A second Chi-square test was conducted to examine age differences in the hypothetical giveaway (Hypothesis 1B). While the majority of both YA and OA indicated they would prefer hand sanitizer over lip balm (YA: 60%, OA: 75%), OA were even *more* likely than YA to select hand sanitizer, *χ*^2^(1, *N* = 296) = 7.38, *p* < 0.01, Ф = 0.16. These results were consistent with our hypothesis and previous pilot study results. In addition, these results are even more compelling in the current context, because hand sanitizer was out of stock at many stores at the time of data collection (see Figure 3B).

##### Age Differences in the Fear of COVID-19 (Hypothesis 2)

To test for age differences in the fear of contracting COVID-19, we conducted a univariate analysis of covariance (ANCOVA; using the characteristic variables that showed age differences in Table 1 as covariates) comparing fear of contraction scores between YA and OA. Contrary to our prediction that OA would report greater fear of contracting the virus than YA, there were no significant age differences in this variable, *F*(7, 327) = 0.98, *p* = 0.45. On average, both YA (*M* = 5.73, *SD* = 2.17) and OA (*M* = 5.41, *SD* = 2.01) rated their fear of contraction slightly above the midpoint of the 10-point scale (Figure 4B).

##### Age Differences in Pandemic-Related Behavior Change (Hypothesis 3)

Another univariate ANCOVA was conducted to compare YA and OA on the pandemic-related behavior change variable and was found to be statistically significant, *F*(7,335) = 7.94, *p* < 0.01, *η*_p_^2^ = 0.15. OA reported more behavior change (*M* = 7.91, *SD* = 0.97) than YA (*M* = 7.25, *SD* = 1.17). These results are consistent with our hypothesis (Figure 5B).

##### Media Exposure Related to Fear (Hypo3thesis 4)

To investigate whether each age group preferred one media source over another, we conducted a Wilcoxon test for paired samples separately for each age group. YA engaged with social media outlets (*M* = 4.37, *SD* = 0.79) more frequently than news media outlets (*M* = 3.38, *SD* = 0.96; *Z* = −8.54, *p* < 0.001). Conversely, OA engaged with news media outlets (*M* = 4.05, *SD* = 0.84) more frequently than social media outlets (*M* = 3.39, *SD* = 1.38; *Z* = −5.25, *p* < 0.001).

Next, we investigated the relationship of media exposure with fear of contracting the coronavirus using a Spearman correlation analysis (Hypothesis 4). We found a positive relationship between the frequency of engaging in social media and the fear of contraction, *ρ* = 0.19, *p* < 0.01. We also found a positive relationship between the frequency of engaging in news media and fear of contraction, *ρ* = 0.23, *p* < 0.01. These results were consistent with our hypothesis that more media engagement would be associated with an increased level of fear of contracting the coronavirus.

##### Media Engagement as a Moderator of Fear (Hypothesis 5)

In hypothesis 5, we predicted that news media engagement would moderate the age differences in fear of contraction. Specifically, we hypothesized that news media engagement would be a key moderator in this model. We conducted a moderation analysis incorporating both media types (social and news media) as moderators, while including the covariates that showed age differences in our sample using Hayes’ PROCESS macro for SPSS statistical software, Model 2 (Hayes, 2013). In this model, we found no significant interactions of our media engagement variables on the relationship between age and fear of contraction. Although we found that the moderation model was significantly predictive [*F*(3, 330) = 4.37, *p* < 0.001, *R*^2^change = 0.13], we did not find a significant interaction effect for neither social media engagement [*b* = 0.07, 95% CI (−0.373, 0.517), *t* = 0.32, *p* = 0.75], nor news media engagement [*b* = −0.28, 95% CI (−0.770, 0.203), *t* = −1.15, *p* = 0.25] on the relationship between age and fear of contraction. Thus, neither type of media engagement moderated the relationship between age and fear of contraction; the results did not support our hypothesis.

#### Discussion

The goals of the present studies were to investigate age differences in the consumption of important health-related information, attentional biases inhibiting behavior change, and whether media exposure plays a role in fear and disease-related outcomes. Building on past work, we found supporting evidence that OA are more likely than YA to engage with more positive compared to negative informational materials, depending on the severity of the health event (i.e., Ebola vs. COVID-19). When the health event was less widespread with lower likelihood of contraction (i.e., Ebola), we found that OA preferred to attend to fear-enhancing material more, and had a higher fear of contraction compared to YA. This may be a byproduct of media reporting. During the Ebola outbreak, media was actively reporting the coverage in the local Ohio news. OA tend to watch more news compared to YA (Mares and Woodard, 2006), which may lead to OA reporting greater fear of contracting Ebola compared to YA. In the COVID-19 pandemic, both age groups may have been engaged in media exposure more equally, resulting in a lack of age effects as shown in the COVID-19 study. Specifically, we noticed that the lack of age effects in the COVID-19 study may have been driven more by YA reporting greater fear of contraction, at least compared to the Ebola study. We also contributed further evidence that regardless of the attentional biases OA may have, they will participate more in behavior change compared to YA, either in self-reporting behavior change (COVID-19 study) or in selecting items that reduce the spread of disease (both studies). Finally, we contributed to a general literature investigating the relationship between media engagement and emotional outcomes: news media is related to an increased fear of contracting the coronavirus. Social media also showed an association with fear of contraction. These relationships between different types of media engagement and fear of contraction provide an avenue for future research.

##### Positivity Vs. Negativity Biases on Major Health Outcomes

Across our two studies, there were some conflicting results. In the Ebola study, we found that OA preferred fear-enhancing articles compared to YA, but in the COVID-19 study, we showed that both age groups were more likely to choose a fear-reducing rather than a fear-enhancing article, but OA were even more likely to do so. The latter results align with the current literature about positivity effects showing that OA attend more often toward positive and avoid negative material compared to YA (Reed et al., 2014). However, a perplexing finding that YA were still more likely to choose the fear-reducing over the fear-enhancing article in both studies does not align with the negativity bias literature mentioned previously (Reed et al., 2014; Carstensen and DeLiema, 2018). One possible interpretation of these results is the role of social media usage. In our sample, YA scored very high in the frequency of engaging in social media. We investigated whether this high frequency engaging in social media could have influenced the article selection choice for the YA group. Our findings suggest that this was not the case. However, our social media engagement measure was a one-item assessment and does not account for all types of affective media engagement. Perhaps a more sensitive measure of social media engagement would capture the impact of overexposure to disease-related information, which in turn could saturate and influence their consciousness (e.g., promote disengagement with negativity; *message fatigue*; So et al., 2017; Kim and So, 2018). Anecdotally, during data collection for the COVID-19 study, news feeds on social media outlets were flooded with negative news, information, and opinions about the coronavirus pandemic. This is of course speculation, but it could have been possible that YA were over-stimulated with such negative information, promoting disengagement to these affectively negative messages, and thus, YA chose to read an article that was more positive in nature due to the novelty (Kim and So, 2018). This could be a potential avenue to explore in future studies. Researchers should find ways to objectively measure the affective content of news and social media coverage during the time of the major event of interest.

More importantly, we found that although OA prefer positive information over negative, OA still participated in changing their behaviors to prevent illness. In the COVID-19 study, not only did OA show they were more likely to choose receiving hand sanitizer over lip balm, replicating our results from the Ebola study, they also showed a higher level of behavior change due to the threat of COVID-19. Although we interpret the findings from the Ebola study with caution, there are several potential reasons we failed to find age differences in behavior change in the Ebola study. First, the behavior change measure in the Ebola study was a dichotomous scale, which led to limited variability in responses. Second, we had a low sample size in the Ebola study. Third, OA may be responding differentially to the COVID-19 pandemic because older age has been identified as a specific risk factor. In the case of Ebola, the emphasis on contraction was not placed on OA, but rather young children. Nevertheless, the aim of this study was to simply examine whether OA *would* attend to the negative information in these widely publicized events and adjust accordingly. In this case, OA are attending to enough negative information about the severity of major health-related events to make health behavioral adjustments at an even greater rate compared to YA in both studies (through reporting or selection). This relates to potential implications of positivity effects for long-term health (Mather and Carstensen, 2005). Our results mitigate the concern that OA may attend toward positive and away from negative information when negative information is most advantageous for their health (Reed et al., 2014). We also succeeded in our pursuit to provide a non-experimental investigation and test positivity effects within a contextually relevant scenario, which are recommendations for future research in the positivity effects literature (Reed et al., 2014).

Our findings for the COVID-19 study were consistent with positivity effects found in studies on decision making in health-related decision strategies (Löckenhoff and Carstensen, 2007). It appears that when OA make important health-related decisions, an attention toward positive over negative information becomes the primary strategy (Löckenhoff and Carstensen, 2007; Isaacowitz and Choi, 2012). It is possible that because the Ebola outbreak was quite fresh during the days of data collection and older age was not indicated as a risk factor, OA may have preferred to attend to the negative news article to gather information. Nonetheless, when the threat of contraction was more prevalent (e.g., COVID-19), the motivation to attend to positive information to prioritize emotional goals may be perceived as most beneficial for the OA population (Löckenhoff and Carstensen, 2004; Isaacowitz and Choi, 2012). These results suggest that OA may engage in emotion regulation (i.e., by selecting to attend to fear-reducing information vs. fear-enhancing) when OA are the more threatened group. Ebola results aside, the results from the COVID-19 study regarding article selection provides evidence for the Cognitive-Functional Model for the Effects of Discrete Negative Emotions on Information Processing, Attitudes, and Recall (Nabi, 1999). This theory posits that individuals will be less motivated to engage with messages if they experience emotions with avoidance tendencies (e.g., fear). If applicable, individuals will consider cues related to alleviating negative affect (e.g., attend toward a more positive message).

Along with explanations of motivational goals, there may be a neurological explanation for these positive preferences. The aging-brain model posits that positivity effects occur with age due to the degeneration process of the amygdala inhibiting affective responses to negative information (Cacioppo et al., 2011). In this explanation, it may be possible that OA are not processing negative information in the same way as YA do, leading to the preference for positive information. Although there is more research to conduct, and our study did not measure brain activation, this explanation does not align across our two studies. Specifically, within the YA group, YA preferred the fear-reducing articles across both studies. In the Ebola study, we also found OA preferred the fear-enhancing article. Future work should include brain imaging to address the role of brain aging in a health decision-making context. There is a need for neurobiological studies that properly assess the aging brain and how this process influences positivity effects (Lighthall, 2020).

Our work was also consistent with Isaacowitz and Choi (2012) in finding both a positivity effect, with concomitant increase in health behavior change. Like Isaacowitz and Choi, these results provide converging evidence that OA may allocate their cognitive resources in a more flexible manner, such that health-protecting information is highlighted without impacting mood and well-being. Further, it appears that the information that is utilized promotes more healthful habits, something that is especially valuable given that OA are an at-risk population.

The findings in both the Ebola and COVID-19 study differed from what was found in the study conducted by Barber and Kim (2020). In our studies, OA showed a greater fear of contraction, and in the COVID-19 study, OA reported greater pandemic-related behavior change. We speculate that the differences in findings between our investigations and those of Kim and Barber is likely due to the differences between the time of data collection in the course of the pandemic and how we measure worry/fear. But these nuances will be important to study in future work.

We were not the first to examine positivity effects using a more ecological approach (Ready et al., 2007; Schryer and Ross, 2014; Ford et al., 2018). For instance, Ford et al. (2018) examined age differences in positivity effects on memory for the 2013 Boston Marathon bombings shortly after the event and 6 months post-event. By using the traumatic event as a stimulus to investigate positivity effects, the authors were able to explore age by time interactions in memory for real events. OA were more likely than YA to report focusing on the positive aspects immediately after the event, with no age differences in focusing on the negative aspects. Over the 6-month period, the authors found that YA increased their focus and memory toward the negative aspects, while OA decreased their focus on the negative components. We aimed to use naturally occurring fear-related events (i.e., Ebola and COVID-19) to assess age differences in fear and relate this to health-related outcomes, especially because it is difficult to induce fear using emotion-provoking stimuli (e.g., film clips) in a lab setting (Stanley and Isaacowitz, 2015). We believe we succeeded in this goal and hope future research can further explicate the impact of positivity effects in naturally occurring events.

#### The Relationship of Media Engagement on Emotions

We were also interested in the role of media engagement on one’s level of fear, specifically during a fearful event. Greater engagement with news and social media were related to a higher fear of contraction. These results align with the literature on mass media and the fear of crime and other threats (e.g., terrorism; Heath and Gilbert, 1996; Nellis and Savage, 2012). Mentioned in the review by Heath and Gilbert, investigating mass media as an effect on an outcome is not simply a main effect, but rather, researchers should attend to nuanced characteristics (e.g., moderating factors) that play a role in fear and media engagement. In our Ebola study, we did not measure media engagement at all. In the COVID-19 study, we measured media engagement, but not to the extent where we could extract some of these more nuanced details (e.g., specific network or social media outlet used, the affective content of the media outlets used, one’s personal perception of how credible each media outlet is that they engaged with, etc.). It will be important for future work to capture some of these details to understand the relationship between media engagement and fear.

Although OA may watch news media more often compared to YA (Mares and Woodard, 2006), we did not find that media engagement affected age differences in the fear of contracting COVID-19. However, there still may be evidence that media engagement can influence levels of fear. There may be a fine line between receiving important health information and over consumption of news media information that results in an increased fear of an event. Eliciting fear is not necessarily always harmful. In fact, messages that have a strong fear appeal coupled with high efficacy have been shown to produce the greatest levels of behavior change for public health campaigns compared to campaigns with lower fear appeal and efficacy (Witte and Allen, 2000). Although our findings from the Ebola study are tentative due to the small sample size, comparing our results with the COVID-19 study may shed light on the findings of Witte and Allen. In the Ebola study, we found that OA feared contracting Ebola more compared to YA, but in the COVID-19 study, we found no age differences. It may be possible that because Ebola was such a localized and acute event in the United States there was less fear appeal (Witte and Allen, 2000). In contrast, because COVID-19 media coverage was so extensive and prolonged, both YA and OA were equally exposed to a high amount of information (i.e., high fear appeal) from media outlets and provided with health protective instructions to avoid contracting COVID-19 (i.e., high efficacy). In future studies, researchers should think about these media factors and attempt to objectively measure these variables. Unfortunately, we did not account for these factors in the current studies.

Although we did not find that the relationship between age and fear of contraction depended on media exposure, there still may be a moderating model for future investigations, especially given the lack of relationship between age and fear of contraction. For instance, Jung (2014) examined the moderating effects of media exposure on the relationship of socioeconomic position (SEP) and cancer worry. Jung found that this relationship between SEP and cancer worry depended on the levels of media exposure, but health-specific media exposure had more consistency compared to general media exposure. Therefore, future studies should aim to investigate both media types with more explanatory details, as well as incorporating other media outlets specifically (e.g., examining each social media program separately) to further understand how mass media information affects emotions.

### Limitations

This study is not without limitations. First, our media engagement measures were one item. In future studies, it would be advantageous to create an inventory of media engagement to provide multiple items with high reliability. Another limitation was that we were unable to collect data for the COVID-19 study in a lab setting. The study might have been improved if we were able to collect data in the lab, specifically for our article selection and item giveaway. In our Ebola pilot study, participants were able to select a news article and read the entirety of the article, as well as physically select a giveaway prize at the end of the study. However, intentions have been shown to be related to behaviors (Webb and Sheeran, 2006), suggesting that participants’ hypothetical responses likely relate to how they would behave if actually faced with the choice. Another limitation is that we focused on only one negative emotion: fear. It is possible that different discrete emotions or moods (e.g., worry, stress, and anxiety) are differentially related to behavioral changes for young and older adults. For example, a functional approach to emotions highlights the unique action tendencies associated with each discrete emotion (Frijda, 1986). We chose to study fear in this research because it seemed highly related to the context of disease outbreaks, and from a functionalist account, fear is associated with the urge to avoid or escape. Future research should investigate other negative emotions to determine whether these relationships hold for all negative emotions.

### Conclusion

These studies add to the existing literature of fear and aging, age differences in attention to health-related information and behavioral change, and media influences on health-related outcomes. Although positivity effects were still observed in the context of disease outbreaks, we also found that OA were more likely than YA to engage in protective health behaviors. We found that depending on the severity of the health-related event, results may vary. Specifically, the at-risk group may respond differently to specific emotional outcomes (i.e., fear of contraction, positivity effects).

Future studies should examine how fear relates or causes behavioral change in other health-related instances, especially among OA. We were able to collect data during a fear-provoking event, but future research should examine more controlled ways to elicit fear effectively. In addition, we chose to examine the emotion of fear as it relates to contracting an illness or disease. Future work should incorporate other emotions to help further understand the general effects of emotion on feelings related to a contextual event. We believe that understanding how age differences in emotions play a role in behavioral change is vital in future intervention and preventative programs/research. This research showed that although OA may avoid negative information about an important health-related event, they are effective at extracting the information needed to participate in health protective behaviors. The good news is that regardless of whether positivity effects were observed, OA still reported adequate health protective behaviors, suggesting that affective preferences did not interfere with these important health-protective behavior changes.

## Data Availability Statement

The datasets presented in this study can be found in online repositories. The names of the repository/repositories and accession number(s) can be found at: https://osf.io/ynbm3.

## Ethics Statement

The studies involving human participants were reviewed and approved by the University of Akron Institutional Review Board (IRB# 20200403). All participants provided informed consent before participation. The patients/participants provided their written informed consent to participate in this study.

## Author Contributions

All authors contributed to the design, conception, and completion of the study. AV and JS were responsible for providing the theoretical and methodological plan of the study. AV took lead in the data analysis and interpretation along with MV and JS. AV wrote the first drafts of the manuscript with supporting contributions from MH, JS, and JT. The manuscript was revised and modified by MV, JS, and JT. All authors contributed to the article and approved the submitted version.

### Conflict of Interest

The authors declare that the research was conducted in the absence of any commercial or financial relationships that could be construed as a potential conflict of interest.

## Footnotes

^1^All except hypothesis 1B were pre-registered in the Open Science Framework for the COVID-19 study. Hypothesis 1B was accidently left out upon registering. However, we intended this hypothesis to be pre-registered, especially since we included the same hypothesis in the previous Ebola study. Besides hypothesis 1B, the other hypotheses align with 1, 2, 3, 5c, and 6c in the pre-registered document, respectively. The remaining pre-registered hypotheses are addressed in different manuscripts.

^2^https://osf.io/ynbm3

^3^https://osf.io/K6X8N/

^4^https://osf.io/347F8/
